# Hamilton depression rating scale and montgomery–asberg depression rating scale in depressed and bipolar I patients: psychometric properties in a Brazilian sample

**DOI:** 10.1186/s12955-015-0235-3

**Published:** 2015-04-02

**Authors:** Adriana Munhoz Carneiro, Fernando Fernandes, Ricardo Alberto Moreno

**Affiliations:** Mood Disorders Unit (Grupo de Disturbios Afetivos- GRUDA), Department and Institute of Psychiatry, School of Medicine, Universidade de São Paulo (USP), Dr. Ovídio Pires de Campos St., 785 – 3rd floor -Ala Norte, Cerqueira César, São Paulo, SP Post code: 05403-010 Brazil

**Keywords:** Validity, Reliability, Rating scales, Bipolar disorder, Depression

## Abstract

**Background:**

The Hamilton Depression Rating Scale (HAM-D) and the Montgomery–Asberg Depression Scale (MADRS) are used worldwide and considered standard scales for evaluating depressive symptoms. This paper aims to investigate the psychometric proprieties (reliability and validity) of these scales in a Brazilian sample, and to compare responses in bipolar and unipolar patients.

**Methods:**

The sample comprised 91 patients with either bipolar I or major depressive disorder from a psychiatric institute at São Paulo, Brazil. Participants were recruited and treated by clinicians through the Structured Interview for DSM-IV criteria, and had previously been interviewed by a trained, blind tester.

**Results:**

Both scales indicated good reliability properties; however, the MADRS reliability statistics were higher than those of the HAM-D for detecting initial symptoms of unipolar depression. Correlation between the tests was moderate. Despite demonstrating adequate validity, neither test achieved the levels of sensitivity and specificity required for identification of a cutoff score to differentiate bipolar I and unipolar patients.

**Conclusions:**

Both scales demonstrate adequate reliability and validity for assessing depressive symptoms in the Brazilian sample, and are good options to complement psychiatric diagnosis, but are not appropriate for distinguishing between the two affective disorder types.

## Background

The general term depression can be applied to a wide range of states, and is defined by symptoms that can be present in a number of different clinical or psychiatric conditions, associated with the use of psychoactive drugs, or even manifest under normal conditions (such as grief or sadness) [1]. By contrast, patients with major depressive disorder present with a group of consistent symptoms that occur throughout most of the day, almost every day, for at least 2 weeks [2].

As is the case for certain other psychiatric disorders, specific biological markers are absent for mood disorders, which means that diagnosis tends to be based on the presence of a syndrome, i.e., a group of relatively stable or recurrent signs and symptoms. Furthermore, some symptoms can be confounded with physical disorders, such as sleep disturbances and changes in weight or libido; these complicating factors facilitate underdiagnosis and confusion with bipolar disorder [3-7].

Consequently, the use of clinical rating scales is required to improve diagnosis quality, to reduce bias caused by physical symptoms, to assess prognosis during treatment, and to evaluate outcomes [8,9]. A large number of scales for evaluating depressive conditions are available, but the Hamilton Depression Rating Scale (HAM-D) [8] and the Montgomery–Asberg Depression Rating Scale (MADRS) [10] are the most common rating scales in use, and are commonly applied to establish clinical criteria for distinguishing levels of severity and for measuring evolution of and recovery from a depressive episode [1].

Despite the differences in content, form of scaling and number of items, most studies that compare HAM-D and MADRS have indicated that the scales are correlated. Some studies reveal correlation coefficients between 0.65 and 0.94 [9,11-13], which suggests that the two instruments may actually measure slightly different aspects of depression. For this reason, we will describe the instruments in sequence.

The HAM-D was developed for administration with patients previously diagnosed with affective disorder, as a measure to quantify the results obtained during the clinical interview, but it has largely been used to assess the efficacy of antidepressive treatment [8,14]. Considered the “gold standard” of depression measures, the HAM-D serves as a point of reference for more recently developed scales. The HAM-D is a hetero-rated scale: it requires a trained rater with sufficient knowledge of the instrument and the symptoms of the depressive syndrome. In order to ensure fidelity, a standardized interview guide was created as part of the training [11,15].

Since first published by Hamilton [8], the scale has been largely applied, and many studies of it have been performed. Bagby et al. [11] used the MEDLINE database to access and review studies that used the 17-item version and that were published between the instrument’s original release in 1979 and 2003. Results indicated good internal, interrater, and retest reliability estimates for the overall scale, but weak interrater and retest coefficients at the item level. As expected, evidence for good convergent, discriminant, and predictive validity was also found. Other studies have found coefficients ranging from 0.83 to 0.94. Interrater reliability of the scale proved consistent, exceeding 0.85 [16]. Nonetheless, the authors highlight some deficits of the scale, mainly due to Hamilton’s conception of depression, which was created more than 40 years ago.

In Brazil, there are few studies about the psychometric proprieties of the HAM-D. The first study with the HAM-D-17 was conducted in the 1980s. In 1987, Dratcu, Ribeiro and Calil [17] assessed severity of patients’ depressive levels with the HAM-D and compared these results with those of the MADRS. Results indicated a moderate correlation between the HAM-D and the MADRS, and low sensitivity of the HAM-D-17 for assessing severity of symptoms. Carvalho [18] designed a translation study, using the HAM-D with 63 bilingual undergraduate students; and Fleck et al. [19] examined the factorial structure of the HAM-D in 130 depressed inpatients from France and Brazil. The authors found differences in the distribution of the factor solution, such that the Brazilian solution indicated four factors, one more than in the French version, as well as differences in the way anxiety items were distributed. No existing reliability studies with Brazilian samples were found for the HAM-D or the MADRS.

The MADRS items are designated to remedy the psychometric limitations of the HAM-D and to measure change during treatment. The scale’s main advantage is its focus on core symptoms to the exclusion of somatic and psychomotor items. The MADRS has been translated into more than 24 languages. Reliability analyses have confirmed the ability to discriminate changes during treatment; interrater reliability coefficients have ranged from 0.89 when with a psychiatrist and a general practitioner and applied on a Swedish sample that lives on North America. Results indicated a reliability of 0.95 when applied by American researches, 0.97 Swedish researchers, 0.97 with a Swedish and an English applicator, and 0.93 with an american psychiatrist with a nurse [10,20]. Zimmerman et al. [21] conducted a literature review of the MADRS to verify the cutoff for defining remission, using MEDLINE data from 1966 to 2002, resulting in ten studies. According to them, ≥4 was the best cutoff score to delineate significant symptoms of depression; whereas the majority of the literature defines an optimal cut off < 9. Zimmerman et al. [21] recommend caution in interpreting their data, however, because not all studies used the same inclusion criteria, and not all patients were being assessed for major depressive disorder (MDD). The previously mentioned work by Dratcu, Ribeiro and Calil [17] is the only Brazilian study on the MADRS.

Both scales have ample psychometric evidence that indicates they are valid measures of symptom outcome in major depression. Validity studies are primary conditions to a test, considering that ensures the degree to which an empirical evidence is supported [22,23].

Validity is often treated as a unitary concept, but the process of validation should be an ongoing process of accumulating various kinds of evidence [23]. Where translation of an instrument is concerned, the first form of evidence relates to content. A literal translation does not ensure that the test measures the same constructs as the original instrument did. Results obtained by Fleck et al. [19] comparing French and Brazilian samples demonstrated the importance of using appropriate statistical analysis, such as factor analysis, to examine and avoid translation threats. Variations in depression scores with the HAM-D have been reported in other studies, indicating the importance of considering cultural differences when validating scales [24,25]. Other important and common evidence is related to other variables, that address the degree that measure are consistent with the construct, as in the study by Dractu et al. [17]; however, this study was performed more than 20 years ago, underscoring the dearth of Brazilian studies despite the scales’ being the most commonly used worldwide.

As was mentioned earlier, no reliability study with a Brazilian sample was found. Reliability is an important component of verifying whether a test is able to measure the proposed construct; i.e., the scale should yield similar answers about the same or similar constructs [26]. In the case of the HAM-D and the MADRS, such reliability is particularly important for ensuring efficacy of clinical drug trials. Within this context, the present study aims to explore the psychometric properties (validity and reliability) of the HAM-D-17 and the MADRS. Secondarily, it aims to compare responses of bipolar I and unipolar patients.

## Methods

### Participants

The sample comprised 91 adult patients experiencing a major depressive episode: 39 (43%) diagnosed according to APA criteria [27] with MDD and 52 (57%) with bipolar disorder type I. All participants were 18 to 59 years old (M = 33.74; SD = 12.10); 63.4% (n = 59) were female. Participants were selected using a version of the *Structured Clinical Interview for DSM* (SCID) [28], which was administered by trained psychiatrists. Exclusion criteria were: presence of psychotic symptoms; comorbidities with other psychiatric disorder; and uncontrolled clinical diseases, such as hypothyroidism, diabetes, or hypertension; and who were receiving other psychiatric treatment.

### Instruments

The Structured Clinical Interview for DSM-IV Axis I Disorders – Clinical Version (SCID-CV) [28] was developed to standardize the psychiatric diagnostic procedures based on interview; the version that had been translated into Portuguese was utilized in the present study. The reliability study was performed in psychiatric patients from a hospital situated in São Paulo’s interior. Test-retest methodology was employed using a two-day interval between interviews. Forty-five (45) patients, predominantly female (60%), with a mean age of 34.9 years (SD = 11.8) participated. The degree of concordance for the diagnosis (Kappa) was 0.83, demonstrating that the instrument had good reliability.

Although the HAM-D [8] is regarded as the “gold standard” for assessing severity of depressive episodes in patients with mood disorders, it was not originally designed to be a diagnostic instrument for depression. Carvalho [18] translated the questionnaire version for use in Brazil by administering the back-translated version to 63 bilingual university graduates. The 17-item version was employed in the present study. In this version, each item is scored from 0 to 2 or from 0 to 4; total scores can range from 0 to 52. The HAM-D scale was not originally designed with cutoff points to designate levels of severity of the depressive condition; therefore, we used Blacker’s research [29] to define cutoff points and severity levels as follows: > 23 = very severe; 19–22 = severe; 14–18 = moderate; 8–13 = mild; and < 7 = remission. The scale predominantly assesses cognitive and vegetative symptoms, with relatively few items related to social, motor, anxiety, and mood factors. Results are categorized as mild, moderate, or severe depression. Reliability of the instrument is based on non brazilian studies, and lies in the 0.83–0.94 range [16].

The MADRS [10] is a 10-item semi-structured scale specifically designed to indicate the severity of the depressive condition. The validation study for use in Brazil was carried out by Dractu et al. [17], who compared the scale against the HAM-D and the Visual Analog Mood Scale in 40 depressed patients. The MADRS assesses mood symptoms exhibited over the preceding 2 weeks, scoring items from 0 to 6, to give a maximum total score of 60 points. Müller [30] proposed the following cutoff points for severity: 0–8 = remission; 9–17 = mild; 18–34 = moderate; and >35 = severe. The items assess somatic, cognitive, vegetative and anxious symptoms. The inter-item reliability of the instrument in an international study was 0.86 [31].

### Procedures

Patients were recruited from the Institute of Psychiatry, located at the Clinical Hospital of the University of São Paulo, Brazil, School of Medicine, after approval of ethic committee CAPPesq (CAAE: 02222012.8.0000.0068). They were diagnosed according to the DSM-IV TR [27]. They were included in the study only after they had read, understood, and signed the Informed Consent Form. Participants were allocated randomly to one of two blocks via a sequence generated by a biostatistician, and were assessed individually by a blind tester trained to administer the scales. The scales took an average of 1 hour to administer. The patients were assessed with the scales prior to treatment (V0), and at four (V4) and eight (V8) weeks after start of treatment, according to the LIthium and CArbamazepine compared to lithium and VALproic acid in the treatment of young bipolar patients (LICAVAL) study [32].

### Statistical analysis

The reliability of the HAM-D-17 was determined on the following basis: (1) interrater reliability, calculated by the intraclass correlation coefficient; (2) internal consistency, calculated using Cronbach’s alpha. Validity was assessed as follows: (1) sensitivity and specificity among groups, using the Receiver Operation Characteristic (ROC) curve; (2) correlation between the HAM-D and the MADRS, using Spearman’s rank correlation; and (3) mean differences in response to MADRS and HAM-D items at V0, calculated by the Mann–Whitney test. All significance tests used *p* < .05 as the minimal criterion.

## Results

The internal consistency results for the HAM-D-17 total, according to Cronbach’s Alpha (*α*) were α =0.83 (V0), α =0.71 (V4) and α =0.85 (V8), whereas the MADRS statistics were α =084; α =0.70 and α =0.80, respectively, showing excellent reliability of both instruments at first and last assessment and good reliability at V4 [26]. Results for the intraclass correlation coefficient are given in Table 1.

**Table 1:** **Intraclass correlation coefficient of HAM-D-17 and MADRS for total sample (n = 91), bipolar I (n =52), and unipolar (n =39) patients**

**Table**	**HAM-D-17**			**MADRS**		
	**V0**	**V4**	**V8**	**V0**	**V4**	**V8**
TOTAL	0.80 (0.73–0.86)	0.71 (0.60–0.80)	0.85 (0.79–0.90)	0.84 (0.78–0.89)	0.70 (0.58–0.80)	0.80 (0.71–0.87)
Bipolar	0.78 (065–0.87)	0.73 (0.58–0.85)	0.63 (0.41–0.80)	0.75 (0.62–0.86)	0.79 (0.68–0.89)	0.73 0.55–0.85)
Unipolar	0.83 (0.74–0.90)	0.70 (0.51–0.83)	0.85 (0.76–0.92)	0.86 (0.76–0.92)	0.85 (0.78–0.92)	0.83 (0.72–0.91)

CI =95%.

**Figure 1:**
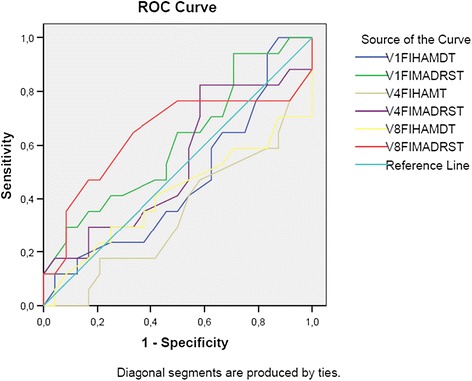
**ROC curve for bipolar I (n =52) and unipolar (n =39) patients at V0, V4, and V8.**

Table 1 shows that the intraclass coefficient of the scales ranged from 0.70 to 0.85, and that reliability declined at V4 in both groups for both scales. In the bipolar I group, a reduction in reliability on the HAM-D-17 with increased weeks of treatment was observed, while on the MADRS, this was evident only in comparison of V0 with V8. According to the reliability parameters adopted, scores over 0.60 indicate adequate reliability. Therefore, all scores qualified as good or excellent [26]. The ROC curve was plotted to investigate the sensitivity and specificity among groups and determine a possible cutoff point (Figure 1).

Results obtained with the ROC curve indicated that the area under the curve for all the variables ranged between 0.34 and 0.64, i.e., below the minimum criterion stipulated for differentiation (0.70). In addition, the curve indicated no definable cutoff score for discriminating symptoms of bipolar I patients from those of unipolar patients.

Group differences in responses to items at time of entry for treatment (V0) were assessed using the Mann–Whitney U test. Results are presented in Table 2.

**Table 2:** **Differences in mean scores on the HAM-D-17 between bipolar I (n = 52) and unipolar (n = 39) groups at time V0**

	**DIAGNOSIS**	**N**	**Mean rank**	**Sum of ranks**	**U**	***z***	p*
1. Depressed mood	Bipolar	39	33,95	1324,00	544	−1.917	0.055
	Unipolar	37	43,30	1602,00			
2. Guilt	Bipolar	39	37,10	1447,00	667	−0.613	0.540
	Unipolar	37	39,97	1479,00			
3. Suicide	Bipolar	39	37,19	1450,50	671	−0.619	0.536
	Unipolar	37	39,88	1475,50			
4. Insomnia early	Bipolar	39	36,96	1441,50	662	−0.690	0.490
	Unipolar	37	40,12	1484,50			
5. Insomnia middle	Bipolar	39	36,27	1414,50	635	−1.036	0.300
	Unipolar	37	40,85	1511,50			
**6. Insomnia late**	**Bipolar**	**39**	**34,04**	**1327,50**	**548**	−**2.210**	**0.027**
	**Unipolar**	**37**	**43,20**	**1598,50**			
7. Work and activities	Bipolar	39	35,13	1370,00	590	−1.397	0.162
	Unipolar	37	42,05	1556,00			
8. Psychomotor retardation	Bipolar	39	35,26	1375,00	595	−1.458	0.145
	Unipolar	37	41,92	1551,00			
9. Psychomotor agitation	Bipolar	39	41,42	1615,50	608	−1.334	0.182
	Unipolar	37	35,42	1310,50			
10. Anxiety, psychic	Bipolar	39	38,73	1510,50	713	−0.101	0.919
	Unipolar	37	38,26	1415,50			
11. Anxiety, somatic	Bipolar	39	35,96	1402,50	623	−1.082	0.279
	Unipolar	37	41,18	1523,50			
12. Loss of appetite (Somatic symptoms, Gastrointestinal)	Bipolar	38	34,18	1299,00	558	−1.741	0.082
	Unipolar	37	41,92	1551,00			
**13. Somatic symptoms, general**	**Bipolar**	**39**	**32,90**	**1283,00**	**503**	−**2.438**	**0.015**
	**Unipolar**	**37**	**44,41**	**1643,00**			
14. Sexual interest (Genital symptoms)	Bipolar	39	34,05	1328,00	548	−1.955	0.051
	Unipolar	37	43,19	1598,00			
**15. Hypochondriasis**	**Bipolar**	**39**	**34,95**	**1363,00**	**583**	−**1.976**	**0.048**
	**Unipolar**	**37**	**42,24**	**1563,00**			
16. Loss of weight	Bipolar	39	38,85	1515,00	708	−0.202	0.840
	Unipolar	37	38,14	1411,00			
**17. Insight**	**Bipolar**	**39**	**43,28**	**1688,00**	**535**	−**3.057**	**0.002**
	**Unipolar**	**37**	**33,46**	**1238,00**			
TOTAL SCORE	Bipolar	38	34,34	1305,00	564	−1.475	*0.140*
	Unipolar	37	41,76	1545,00			

*significant considering 2-tailed curve; CI: 95%.

Of the 17 items in the HAM-D-17, four (**Insomnia late**, **Somatic symptoms–general, Hypochondriasis, and Insight**) revealed significant group differences in mean scores [33,34]. According to mean scores, depressive patients reported more sleep-related difficulties and somatic symptoms and some inappropriate worry about their health in their initial interview than did bipolar I patients, who reported more knowledge about their depressive state state at time of entry. Unipolar subjects had higher overall scores than did bipolar I patients, although this difference was not statistically significant. The same analysis was performed on the results from the MADRS; results are described in Table 3.

**Table 3:** **Differences in mean scores on the MADRS between bipolar I (n = 52) and unipolar (n = 39) groups at time V0**

	**DIAGNOSIS**	**N**	**Mean rank**	**Sum of ranks**	**U**	**z**	p*
**Reported sadness**	**Bipolar**	**39**	**29,12**	**1135,50**	**356**	−**3.880**	**0.000**
	**Unipolar**	**37**	**48,39**	**1790,50**			
**Apparent sadness**	**Bipolar**	**39**	**30,74**	**1199,00**	**419**	−**3.212**	**0.001**
	**Unipolar**	**37**	**46,68**	**1727,00**			
**Inner tension**	**Bipolar**	**39**	**29,76**	**1160,50**	**381**	−**3.669**	**0.000**
	**Unipolar**	**37**	**47,72**	**1765,50**			
Reduced sleep	Bipolar	39	37,94	1479,50	700	−0.241	0.810
	Unipolar	37	39,09	1446,50			
**Reduced appetite**	**Bipolar**	**39**	**31,73**	**1237,50**	**458**	−**3.021**	**0.003**
	**Unipolar**	**37**	**45,64**	**1688,50**			
**Concentration difficulties**	**Bipolar**	**40**	**34,40**	**1376,00**	**556**	−**1.978**	**0.048**
	**Unipolar**	**37**	**43,97**	**1627,00**			
**Psychomotor inhibition**	**Bipolar**	**39**	**28,36**	**1106,00**	**326**	−**4.266**	**0.000**
	**Unipolar**	**37**	**49,19**	**1820,00**			
**Inability to feel**	**Bipolar**	**40**	**30,80**	**1232,00**	**412**	−**3.441**	**0.001**
	**Unipolar**	**37**	**47,86**	**1771,00**			
Pessimistic thoughts	Bipolar	40	36,15	1446,00	626	−1.227	0.220
	Unipolar	37	42,08	1557,00			
Suicidal thoughts	Bipolar	40	35,92	1437,00	617	−1.385	0.166
	Unipolar	37	42,32	1566,00			
**Total score**	**Bipolar**	**40**	**26,79**	**1071,50**	**252**	−**3.172**	**0.002**
	**Unipolar**	**24**	**42,02**	**1008,50**			

*Sig (2-tailed); CI= 95%.

Results of the Mann–Whitney U test showed statistically significant group differences on seven of the 10 items as well as on the total score of the MADRS, with the unipolar group consistently scoring higher. Item-specific comparisons showed that unipolar patients had greater sadness and apparent sadness, tension, psychomotor inhibition, reduced appetite, concentration difficulties, and inability to feel and total score. To conclude, correlation analyses between the scales were performed, in order to determine the degree of similarity of HAM-D and MADRS at the three administration time points. Results revealed correlations of r_*s*_ = 0.78 (*p = 0.01*) at V0, r_*s*_ = 0.84 (*p = 0.001*) at V4 and r_*s*_ = 0.76 (*p = 0.001*) at V8, i.e., excellent levels of correlation [33].

## Discussion

The aim of the present study was to investigate the psychometric properties of MADRS and HAM-D-17 in a Brazilian sample from São Paulo. The results showed good levels of reliability and validity for both scales, indicating that they were able to measure gravity of depressive symptoms. Reliability of the HAM-D ranged from good to excellent at the three administration time points, although few items served to distinguish the more specific symptoms of unipolar depressed subjects. By contrast, 70% of the MADRS items were able to differentiate unipolar depression versus bipolar I depression, and the reliability scores ranged from good to excellent for the three experimental time points.

The reliability of HAM-D-17, as measured by Cronbach’s alpha, was taken from previous data in the literature [8,11]. The mean scores in both groups proved similar to those reported by Rehm, Michael and O’Hara [12], who found statistically significant mean differences on items 10 (Anxiety, psychic), 15 (Hypochondriasis) and 17 (Insights), versus differences found in the present study on items 6 (Insomnia late), 13 (Somatic symptoms), 15 (Hypochondriasis), and 17 (Insight).

Concerning the MADRS, reliability was also as expected, showing scores in agreement with those found in other studies [9,10,21]. As already discussed, most items were able to differentiate unipolar from bipolar I subjects. Despite its extensive use, MADRS lacks published results in Brazil, precluding comparisons against the findings of the present study.

In addition, besides differences among items, it is important to note that the ROC curve failed to indicate a cutoff point for differentiating unipolar from bipolar I depression. It is known that bipolar I patients are often initially diagnosed as unipolar because they tend to seek medical assistance during the depressive phase [4]. For this reason, scales that are sensitive enough to discriminate the two conditions may be promising for assisting diagnosis by health professionals, and should be considered in further studies.

A secondary aim was to compare responses between the unipolar and bipolar I group. Regarding HAM-D score differences, the scale has determined that unipolar depressive patients have a greater tendency to weep and to exhibit more nonverbal expressions of sadness and hopelessness, according to other studies [3,6]. Other results relevant to MDD diagnosis are related to items 7 (work activities) and 8 (psychomotor retardation). Unipolar patients scored higher on these items, although the difference was statistically nonsignificant.

It is also pertinent to highlight some of the observed qualitative differences in depressive and bipolar I symptomology, even those that were not statistically significant. According to the HAM-D results, depressed and bipolar I patients scored similarly on Guilt, Suicide and Psychic anxiety, and Loss of weight. This indicates that both patients frequently entertain ideas of guilt or rumination, and that sometimes these thoughts include the notion that life is not worth living and ideas about the possibility of death, but also excessive fear and concerns with minor matters. It is important to consider that the differences in this kind of ideation were not significant between groups, which is unexpected, considering that greater suicidal ideation is expected for depressed patients [3,7,16]. Additionally, bipolar I patients had a higher (but also nonsignificant) mean score on psychomotor agitation, which is a rating based on observation and indicates motor restlessness. This could be an important nonverbal sign to consider during interviews, and is supported by results from other studies [4-6]. Finally, regarding the statistical difference on item 17 (Insight), it is known that people with bipolar disorder are more likely to seek help when they are depressed than when they are experiencing mania or hypomania [5], so this is an expected result.

Concerning the MADRS, unipolar patients had greater Reported and Apparent sadness, Inner tension, Reduced appetite, Psychomotor inhibition and Inability to feel. It is notable that, of the symptoms listed in Table 3, pessimistic thoughts, Suicide thoughts, and Reduced sleep had higher (but not statistically significant) mean scores among unipolar than bipolar I patients. Considering these results, the MADRS items appear to be more appropriate for assessing unipolar depression, in view that discriminated more significant differences between bipolar and unipolar patients. Finally, a high correlation was found between the two instruments, and was similar to those reported by Dractu, Ribeiro and Calil [17]. Importantly, the sample used by those authors included only unipolar subjects. Müller et al. [14] used a similar sample size (n =40) and found a correlation of (*r*_*s*_ = 0.66; *p > 0.005*). Together with previous data, our findings suggest that the HAM-D and the MADRS are adequate scales for measuring depressive symptoms before and during treatment.

## Conclusions

The Brazilian version of the HAM-D and MADRS scales showed good reliability (coefficients ranging from 0.71 to 0.85) and intraclass correlation, as well as evidence of discriminant and convergent validity (based on the relationship with other variables). The scales proved consistent for assessing depressive symptoms, but limitations in their ability to discriminate unipolar and bipolar I patients were found. This result was expected only for the HAM-D, but was also found for the MADRS. Nevertheless, and consistent with other findings, the MADRS has proven to be a more reliable tool than the HAM-D for assessing depressive symptoms. Further investigations should replicate the present study with larger samples and incorporate scales that may be more sensitive for detecting the different spectrums in order to reduce current limitations.

Moreover, is important to highlight that the MADRS and HAM-D have a number of particularities that should be considered by the clinician, including the number of constituent items and the distribution of symptoms. For instance, the HAM-D-17 contains somatic items that are not included in the MADRS, which for its part, represents a good option in cases where assessment of somatic symptoms of depression is not required, and where comparisons of observed and reported symptoms is desired. Therefore, although the scales constitute useful tools that can help reduce health professionals’ subjectivity, they should be considered as supplemental to rigorous clinical-psychological evaluation.

## Abbreviations

BD: Bipolar disorder
MDD: Major depressive disorder
HAM-D: Hamilton Depression Rating Scale
MADRS: Montgomery–Asberg Depression Rating Scale
ROC: Receiver operating characteristic
